# Decision making for large-scale multi-armed bandit problems using bias control of chaotic temporal waveforms in semiconductor lasers

**DOI:** 10.1038/s41598-022-12155-y

**Published:** 2022-05-16

**Authors:** Kensei Morijiri, Takatomo Mihana, Kazutaka Kanno, Makoto Naruse, Atsushi Uchida

**Affiliations:** 1 Department of Information and Computer Sciences, Saitama University, 255 Shimo-okubo, Sakura-ku, Saitama City, Saitama 338-8570 Japan; 2 Department of Information Physics and Computing, Graduate School of Information Science and Technology, The University of Tokyo, 7-3-1 Hongo, Bunkyo-ku, Tokyo, 113-8656 Japan

**Keywords:** Engineering, Optics and photonics

## Abstract

Decision making using photonic technologies has been intensively researched for solving the multi-armed bandit problem, which is fundamental to reinforcement learning. However, these technologies are yet to be extended to large-scale multi-armed bandit problems. In this study, we conduct a numerical investigation of decision making to solve large-scale multi-armed bandit problems by controlling the biases of chaotic temporal waveforms generated in semiconductor lasers with optical feedback. We generate chaotic temporal waveforms using the semiconductor lasers, and each waveform is assigned to a slot machine (or choice) in the multi-armed bandit problem. The biases in the amplitudes of the chaotic waveforms are adjusted based on rewards using the tug-of-war method. Subsequently, the slot machine that yields the maximum-amplitude chaotic temporal waveform with bias is selected. The scaling properties of the correct decision-making process are examined by increasing the number of slot machines to 1024, and the scaling exponent of the power-law distribution is 0.97. We demonstrate that the proposed method outperforms existing software algorithms in terms of the scaling exponent. This result paves the way for photonic decision making in large-scale multi-armed bandit problems using photonic accelerators.

## Introduction

Machine learning and artificial intelligence have revolutionized information and communication technology. Recently, machine learning techniques based on photonic technologies, known as photonic accelerators, have been studied intensively^1^. Using photonic technologies in machine learning has advantages such as fast and energy-efficient processing^1^. The latest advancements in photonic accelerators include photonic neural networks^2^, photonic reservoir computing^3–6^, coherent Ising machine^7^, optical pass gate logic^8^, and photonic decision making^9–21^.

Photonic decision-making techniques have been used to solve the multi-armed bandit problem, which is fundamental to reinforcement learning^22,23^. In the multi-armed bandit problem, a player aims to maximize the total reward by making a limited number of selections of multiple slot machines (or arms) with unknown hit probabilities. To solve this problem, the player needs to search for the slot machine with the highest hit probability (i.e., exploration). The player then concentrates on the slot machine that is estimated to offer the best chance of reward (i.e., exploitation). However, there is a difficult trade-off between exploration and exploitation, known as the exploration–exploitation dilemma^22^. Excessive exploration leads to a reduction in the total reward, whereas insufficient exploration means that the best slot machine is not identified.

Decision making using photonic technologies has been widely demonstrated^9–21^. For example, chaotic temporal waveforms generated by semiconductor lasers have been used, where the threshold level of the temporal waveform can be controlled^9–12^. Furthermore, mode competition dynamics in a ring–cavity semiconductor laser on a chip has been utilized to solve two-armed bandit problems (i.e., problems with two slot machines)^13^. The lag synchronization of chaos in mutually coupled semiconductor lasers has been used for decision making^14^, and this approach has been extended to laser networks with a large number of slot machines^15,16^. Furthermore, single^17,18^ and entangled^19–21^ photons have been utilized for photonic decision making.

Scalability, in terms of the number of slot machines, is an important challenge in photonic decision making. Although the two-armed bandit problem is the most fundamental problem, extending solutions to large-scale multi-armed bandit problems remains an important challenge. A hierarchical structure using chaotic temporal waveforms was introduced to increase the number of slot machines to 64^10^. In addition, a laser network consisting of coupled semiconductor lasers in a ring configuration has been used to solve problems with up to seven slot machines^15^. However, no solutions have been reported for problems with more than 100 slot machines. In fact, the solutions to large-scale multi-armed bandit problems are expected to exploit the unique advantages of light, such as temporal, spatial, and wavelength domain multiplexing. These solutions are useful in communication applications, such as channel selection^24,25^ and non-orthogonal multiple access (NOMA)^26^.

In this study, we propose a decision-making scheme to solve multi-armed bandit problems using bias control of chaotic temporal waveforms in semiconductor lasers with optical feedback. We numerically investigate the decision-making performance for different numbers of slot machines. Next, we examine the scaling characteristics of the performance in terms of the number of slot machines, up to 1024 machines, which is beyond the number used in the previous studies of photonic decision making. Finally, we compare the performance of our proposed method with that of existing software algorithms.

## Results

### Bias control of chaotic temporal waveforms

**Figure 1:**
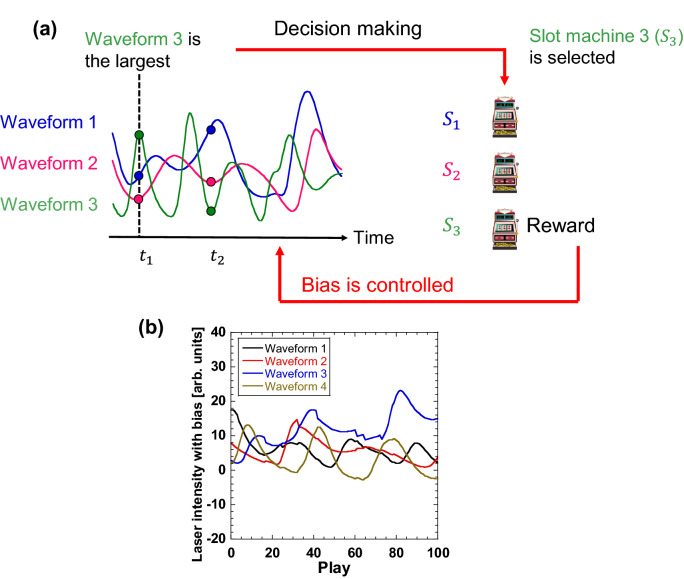
Decision-making method using chaotic temporal waveforms with bias control. (**a**) Schematic diagram. (**b**) Chaotic temporal waveforms assigned to slot machines (*N* = 4).

We consider a multi-armed bandit problem with *N* slot machines, which produce the binary results 1 (hit) or 0 (miss) and have different hit probabilities. Figure 1a shows a schematic of our decision-making method using chaotic temporal waveforms. We numerically generate *N*-independent chaotic temporal waveforms from semiconductor lasers with optical feedback using the Lang–Kobayashi equations^27–29^. Each temporal waveform is assigned to a slot machine, i.e., the chaotic temporal waveform *i* is assigned to slot machine *i* (a total of *N* slot machines). The waveforms are sampled at a constant sampling interval. The bias *B*_*i*_(*t*) is added to the amplitude *I*_*i*_(*t*) of the *i*-th chaotic temporal waveform as:

1 $$\begin{array}{c}{D}_{i}\left(t\right)={I}_{i}\left(t\right)+k{B}_{i}\left(t\right),\end{array}$$

where *k* is the bias coefficient. Slot machine *i*, corresponding to maximum ${D}_{i}\left(t\right)$, is selected by comparing the values of ${D}_{i}\left(t\right)$ for all the temporal waveforms at time *t*. After the selection, bias *B*_*i*_(*t*) is added to (or subtracted from) the amplitude of the chaotic temporal waveform based on the result of the slot machine selection. For example, as shown in Fig. 1a, waveform 3 has the maximum amplitude at sampling time *t*_1_, so slot machine 3 is selected. If the result for slot machine 3 is “hit,” then the bias is added to the amplitude of temporal waveform 3, and the amplitudes of the other temporal waveforms are reduced so that slot machine 3 will be selected more frequently in the future. In contrast, if the result for slot machine 3 is “miss”, the bias is subtracted from the amplitude of temporal waveform 3, and the amplitudes of other temporal waveforms are increased so that slot machine 3 will be selected less frequently in the future. These procedures are repeated by changing the sampling times for the temporal waveforms.

More precisely, the bias *B*_*i*_(*t*) for temporal waveform *i* and slot machine *i* is determined by the tug-of-war algorithm, described by the following equations^30–33^:

2 $$\begin{array}{c}{B}_{i}\left(t\right)={Q}_{i}\left(t\right)-\frac{1}{N-1}\sum\limits_{{i}^{^{\prime}}\ne i}^{N}{Q}_{{i}^{^{\prime}}}\left(t\right),\end{array}$$

3 $$\begin{array}{c}{Q}_{i}\left(t\right)={T}_{i}-\left(1+\omega \right){L}_{i},\end{array}$$

4 $$\begin{array}{c}\omega =\frac{{\widehat{P}}_{top1}+{\widehat{P}}_{top2}}{2-\left({\widehat{P}}_{top1}+{\widehat{P}}_{top2}\right)},\end{array}$$

5 $$\begin{array}{c}{\widehat{P}}_{i}=\frac{{W}_{i}}{{T}_{i}}.\end{array}$$

Here, *Q*_*i*_ is the evaluation value (Q-value) of slot machine *i* in the tug-of-war algorithm, ${\widehat{P}}_{i}$ denotes the estimated hit probability for slot machine *i,* ${\widehat{P}}_{top1}$ is the highest estimated hit probability, and ${\widehat{P}}_{top2}$ is the second-highest estimated hit probability. In addition, *T*_*i*_, *W*_*i*_, and *L*_*i*_ denote the number of total, “hit” (win), and “miss” (lose) selections, respectively, for slot machine *i*.

The bias coefficient *k* is a control parameter for the balance between exploration and exploitation. A smaller bias coefficient leads to finer exploration; however, more time is required to determine the slot machine with the highest hit probability. In contrast, a larger bias coefficient results in a faster transition to exploitation; however, the process could fail to identify the slot machine with the highest hit probability during exploration. Therefore, it is necessary to set an appropriate value for the bias coefficient *k*, which depends on the difficulty of the decision-making problem.

### Decision-making results

As previously discussed, the chaotic temporal waveforms are numerically generated using the Lang–Kobayashi equations^27–29^. The Lang–Kobayashi equations and their corresponding parameter values are described in the “Methods” section, along with an example of the generated chaotic temporal waveforms and their statistical characteristics. Independent chaotic temporal waveforms are generated from different initial conditions for decision making.

First, we consider the multi-armed bandit problem with four slot machines (*N* = 4) with hit probabilities P_1_ = 0.7, P_2_ = 0.5, P_3_ = 0.9, and P_4_ = 0.1. In this setting, slot machine 3 has the highest hit probability. Therefore, selecting slot machine 3 is the best decision. Four independent chaotic temporal waveforms are generated, and each is assigned to a different slot machine. Decision making is performed based on bias control of the amplitude of the chaotic temporal waveforms at a sampling interval of 10 ps. The shortest time taken for one decision-making play is 10 ps, because the sampling interval of the chaotic temporal waveforms is set to 10 ps. However, a specialized post-processing equipment for decision making is required to achieve such a fast decision-making rate.

Figure 1b shows an example of four chaotic temporal waveforms assigned to four slot machines. The chaotic temporal waveforms change, and their amplitudes are updated by adding or subtracting the bias *B*_*i*_(*t*), based on Eq. (1), at each sampling interval. After the 34th play (340 ps), the amplitude of temporal waveform 3 retains the maximum value; hence, slot machine 3 is continuously selected.

**Figure 2:**
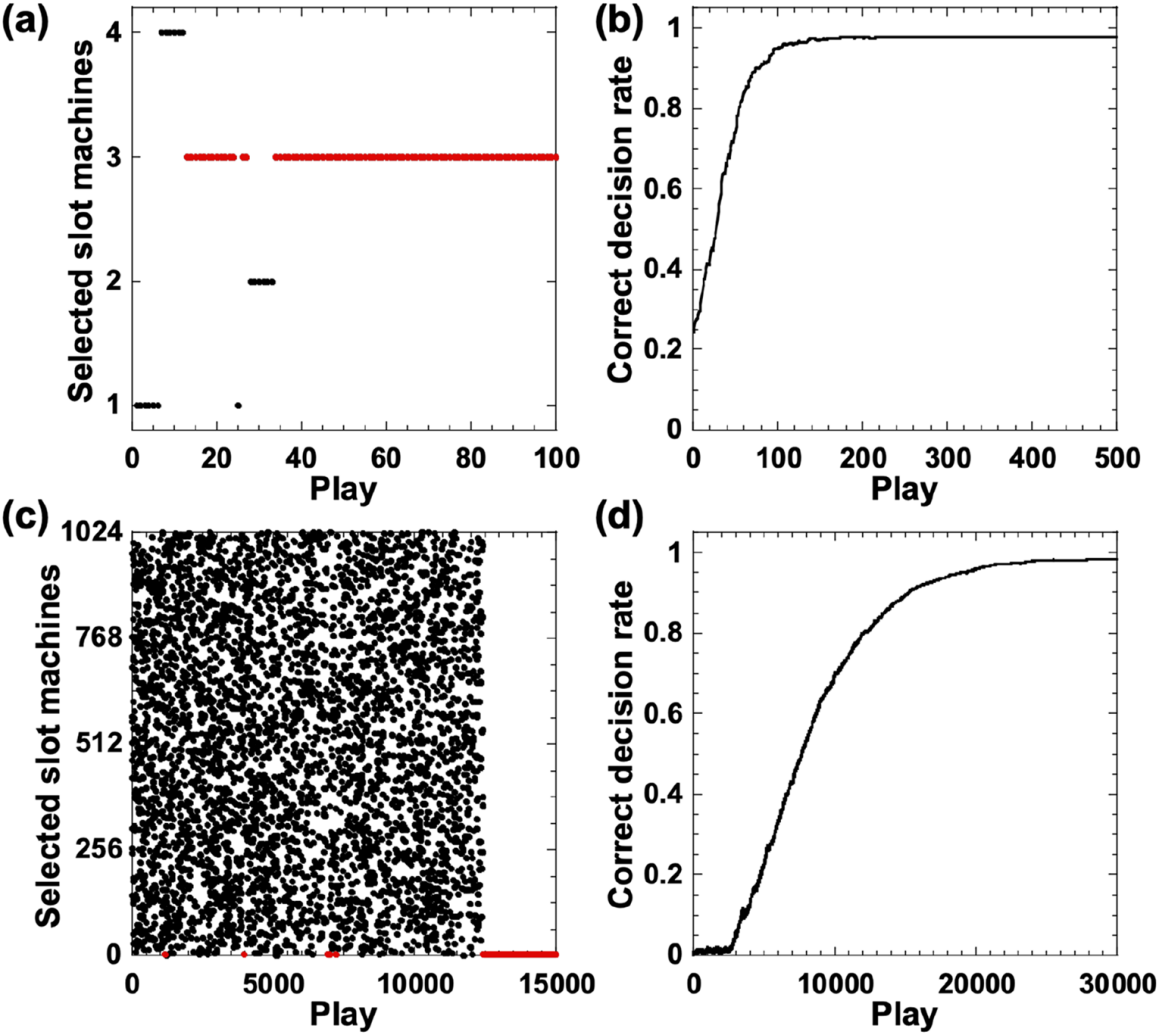
Performance of decision-making process: (**a**) selected slot machines from four slot machines (*N* = 4) (red dots indicates correct selection); (**b**) correct decision rate (CDR) as the number of plays increases for *N* = 4; (**c**) selected slot machines among 1024 slot machines (*N* = 1024) (red dots indicates correct selection); and (**d**) CDR as the number of plays increases for *N* = 1024. The bias coefficients are set to (**a,b**) *k* = 0.3 and (**c,d**) *k* = 1.7.

Figure 2a shows the decision-making process of the chaotic temporal waveforms assigned to four slot machines. One of the slot machines is selected for each play. After the 34th play, slot machine 3 is always selected (the red dots), which is consistent with the maximum value of temporal waveform 3 in Fig. 1b. Therefore, correct decisions are made using the proposed method for this case.

We introduce the correct decision rate (CDR) to evaluate the statistical characteristics of the decision-making performance. This is expressed by the following equation^9^:

6 $$\begin{array}{c}CDR\left(t\right)=\frac{1}{n}\sum\limits_{i=1}^{n}C\left(i,t\right),\end{array}$$

$$t=\text{1,2},\ldots ,m,$$

where $m$ and $n$ represent the numbers of plays and cycles, respectively. In addition, $C(i,t)$ is a function that returns 1 if the slot machine with the highest hit probability is selected, and 0 otherwise, for the *t*-th play and *i*-th cycle. A large CDR indicates that the slot machine with the highest hit probability is selected. We define one cycle as 500 plays ($m$ = 500) and repeat the process for 1000 cycles ($n$ = 1000) to evaluate the decision-making performance. We determine that decision making is correct if the CDR is at least 0.95. Figure 2b shows the CDR for *N* = 4 as the number of plays increase. The CDR reaches 0.95 after approximately 100 plays; thus, the decision making shows high accuracy.

We extend the proposed method to situations involving a large number of slot machines, up to *N* = 1024. In this case, we set the hit probabilities to P_1_ = 0.7, P_2_ = 0.5, P_3_ = 0.9, P_4_ = 0.1,…, P_2*j*−1_ = 0.7, and P_2*j*_ = 0.5 (*j* ≥ 3, where *j* is an integer). In this setting, slot machine 3 has the highest hit probability of P_3_ = 0.9. We also increase the number of plays to $m$ = 30,000 because more plays are required to explore a large number of slot machines and achieve correct decision making. Figure 2c shows an example of the selected slot machines after each play for one cycle for *N* = 1024. Slot machines are randomly and uniformly selected. After approximately 12,500 plays, only slot machine 3 is selected (the red dots). Figure 2d shows the CDR as the number of plays increases for *N* = 1024. The CDR increases gradually and reaches 0.95 after approximately 19,000 plays, determined by the statistical average over 1000 cycles. Therefore, we found that the proposed method can achieve correct decision making, even for a large number of slot machines (*N* = 1024). The number of plays for achieving a CDR of 0.95 depends on the difference between the highest and second-highest hit probabilities. However, the hit probabilities of the other slot machines do not strongly affect the number of plays required to achieve CDR = 0.95.

### Scalability of decision making

**Figure 3:**
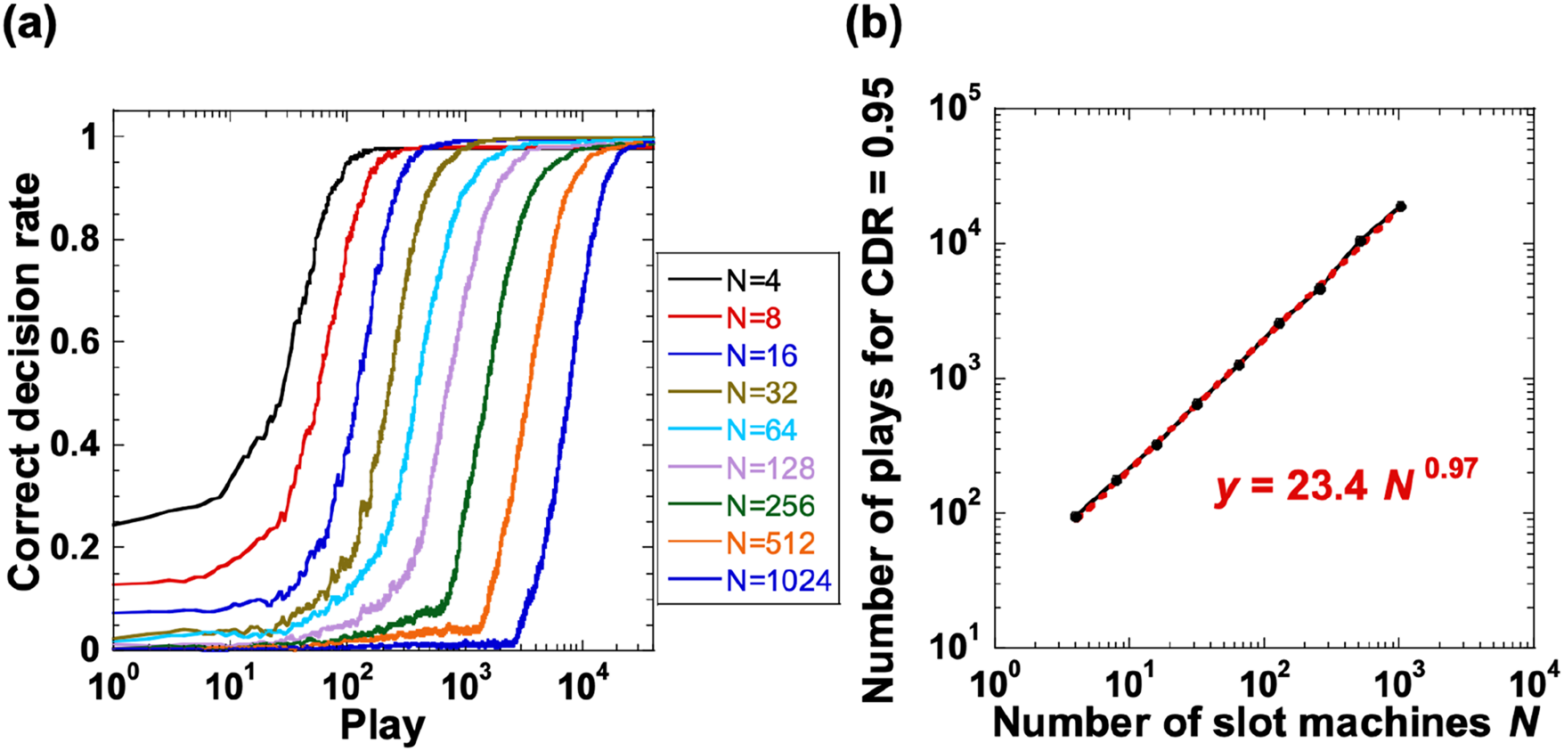
Correct decision rate (CDR) and scaling characteristics. (**a**) CDR as the number of plays increases for different numbers of slot machines from *N* = 4 to 1024 (2^*i*^, *i* = 2, 3,…, 10). (**b**) Relationship between the number of plays *y* at which the CDR reaches 0.95 and the number of slot machines *N* on double-logarithmic scales. The bias coefficient *k* is optimized for different values of *N* (see Fig. 7).

We investigate the scalability of the decision-making performance when the number of slot machines is changed. First, we calculate the CDR for different numbers of slot machines, *N*. We set the hit probabilities to P_1_ = 0.7, P_2_ = 0.5, P_3_ = 0.9, P_4_ = 0.1,…, P_2*j*−1_ = 0.7, and P_2*j*_ = 0.5 (*j* ≥ 3, where *j* is an integer), as shown in Fig. 2. The bias coefficient *k* is optimized for different values of *N*, as described in the “Methods” section. Figure 3a shows the CDR as the number of plays increases for different numbers of slot machines from *N* = 4 to 1024 (2^*i*^, *i* = 2, 3,…, 10). For all values of *N*, as the number of plays increases, the CDR curves gradually increases until they reach 0.95. However, the number of plays required for the CDR to converge to 0.95 increases as *N* increases. It should be noted that the curves are equidistantly distributed on a semi-logarithmic scale; therefore, a scaling law can be obtained from the curves.

As shown in Fig. 3a, we measure the number of plays *y* at which the CDR reaches 0.95 for different values of *N* to investigate the scalability in terms of *N*. Figure 3b shows the relationship between *y* and *N* plotted on a double-logarithmic scale. The number of plays required for CDR = 0.95 shows an approximately linear increase as the number of slot machines increases, as shown in Fig. 3b. We identify a power–law relationship between *y* and *N*, (i.e., *y* = *A N*^*γ*^), and obtain *y* = 23.4* N*^0.97^ from Fig. 3b. The exponent *γ* = 0.97 is close to 1, which indicates that the number of plays required for correct decision making is approximately proportional to the number of slot machines (i.e., order *N*, *O*(*N*)). This exponent is smaller than previous results in other photonic decision-making schemes (e.g., *γ* = 1.16^10^ and *γ* = 1.85^15^).

### Comparison with other decision-making methods

We compare the decision-making performance of our laser-chaos-based method with other decision-making methods. We consider four well-known software algorithms for solving the multi-armed bandit problem: *ε*-greedy^22^, softmax^22^, UCB1-tuned (upper confidence bound 1-tuned)^34^, and Thompson sampling^35^. The hyperparameters are optimized for different numbers of slot machines for the *ε*-greedy and softmax algorithms, whereas there are no hyperparameters for the Thompson sampling and UCB1-tuned methods.

**Figure 4:**
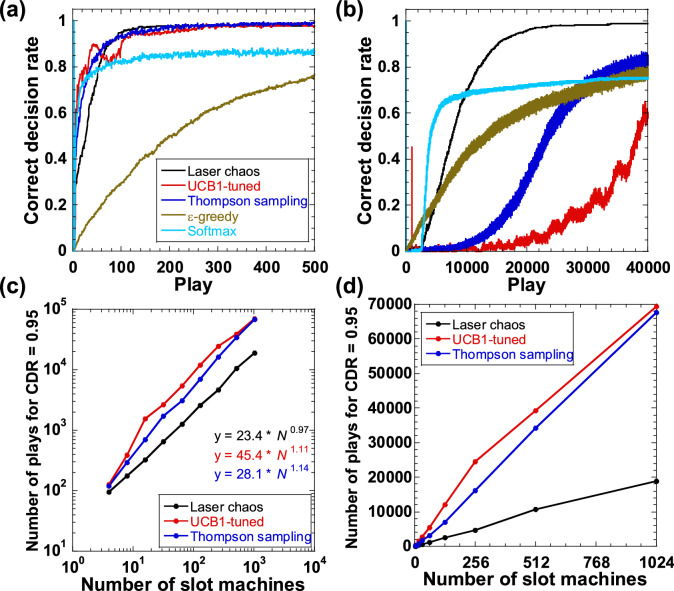
Comparison of correct decision rate (CDR) and scaling characteristics for the laser-chaos-based method (black), the UCB1-tuned (red), Thompson sampling (blue), *ε*-greedy (brown), and softmax (light blue) algorithms. (**a**) CDR as the number of plays increases for *N* = 4; (**b**) CDR as the number of plays increases for *N* = 1024; and (**c**), (**d**) scaling characteristics for the relationship between the number of plays *y* at which the CDR reaches 0.95 and the number of slot machines *N* for laser-chaos-based method (black), UCB1-tuned (red), and Thompson sampling (blue) algorithms on (**c**) double logarithmic and (**d**) double linear scales. The hyperparameter values are optimized for the *ε*-greedy and softmax algorithms for different *N*.

Figure 4a shows the CDR for each play over 1000 cycles for the laser-chaos-based method and the four software algorithms for *N* = 4. The CDR increases and reaches 0.95 for the laser-chaos-based method, UCB1-tuned algorithm, and Thompson sampling algorithm. However, it does not reach 0.95 for the *ε*-greedy and softmax algorithms. The first three methods show similar CDR characteristics, except there are fluctuations in the CDR of the UCB1-tuned algorithm. Next, we increase the number of slot machines to *N* = 1024. Figure 4b shows the CDR for the laser-chaos-based method and four software algorithms with *N* = 1024. In this case, the CDR for the laser-chaos-based method converges above 0.95, whereas the CDR for all four software algorithms does not reach 0.95. A sharp CDR peak appears at approximately the 1000th play for the UCB1-tuned algorithm (the red curve). We speculate that the correct decision making is achieved at approximately the 1000th play for a small number of local explorations. However, the algorithm starts searching the remaining slot machines globally, and hence the CDR decreases again.

We also compare the scaling characteristics of the laser-chaos-based method, UCB1-tuned algorithm, and Thompson sampling algorithm. The number of plays *y* required for the CDR to reach 0.95 with different numbers of slot machines is calculated for each of the three methods. Figure 4c shows the relationship between *y* and *N* for the three methods on a double logarithmic scale. The power-law relationship is approximated, and we obtain *y* = 45.4* N*^1.11^ and *y* = 28.1* N*^1.14^ for the UCB1-tuned and Thompson sampling algorithms, respectively. The exponents for the UCB1-tuned and Thompson sampling, *γ* = 1.11 and *γ* = 1.14, respectively, are larger than that for the laser-chaos-based method, *γ* = 0.97. The small exponent indicates that the laser-chaos-based method performs better than these software algorithms. Figure 4d shows that similar results are obtained using double linear scales for the vertical and horizontal axes, and shows the difference between the methods. For example, when *N* = 1024, the laser-chaos-based method is 3.5 times faster at achieving correct decision making than both the UCB1-tuned and Thompson sampling algorithms. Therefore, the laser-chaos-based method outperforms these well-known software algorithms.

### Effect of temporal correlation on decision making

In the previous scheme, we assigned independent chaotic temporal waveforms to the slot machines for decision making. However, a negative correlation of chaotic temporal waveforms may enhance the decision-making performance^9,36^. Therefore, we now generate correlated chaotic temporal waveforms and assign them to the slot machines. For simplicity, we consider the case of two slot machines (*N* = 2), to which two temporal waveforms with negative or positive correlations are assigned. We generate two identical chaotic temporal waveforms from the same initial conditions, one of which is time-shifted, to obtain two correlated temporal waveforms. The positive and negative correlation values are 0.300 and − 0.583, respectively. We also generate two chaotic temporal waveforms from different initial conditions to obtain independent (non-correlated) temporal waveforms for comparison.

**Figure 5:**
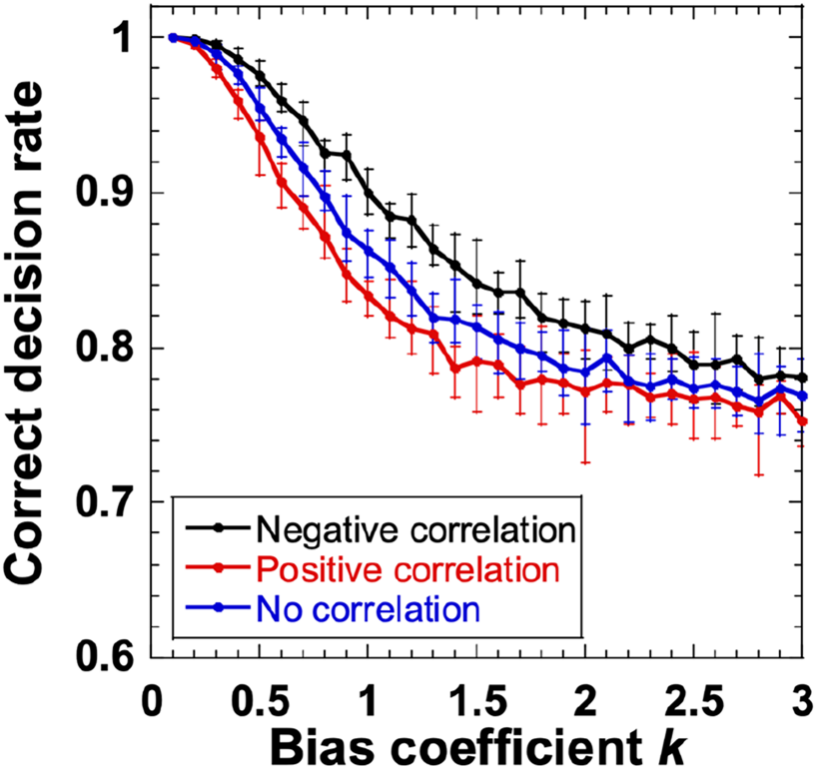
Correct decision rate (CDR) for two slot machines (*N* = 2) assigned to temporal waveforms with negative (black), positive (red), and no (blue) correlations as the bias coefficient *k* increases. The cross bars represent the maximum and minimum values of the CDR for ten repetitions of the numerical calculation.

Figure 5 shows the CDR of the two slot machines (*N* = 2) assigned to the chaotic temporal waveforms with negative, positive, and no correlations, as the bias coefficient *k* is changed. The CDR of the negatively correlated temporal waveforms is larger than that of the independent temporal waveforms. In addition, the CDR of the positively correlated temporal waveforms is smaller than that of the independent temporal waveforms. This indicates that negative correlation is effective for decision making in the case where *N* = 2, because the alternate selection of two slot machines enhances the exploration for the estimation of hit probabilities.

However, this effect is only observed for a limited range of the bias coefficient *k*, and the CDR is between 0.8 and 0.9. When *k* is optimized, there is no major difference between the CDR of the negative-, positive-, and non-correlated temporal waveforms. In addition, when *N* is large, the benefits of correlation disappears because different correlations emerge among *N* temporal waveforms, and the *N* temporal waveforms effectively become independent.

In addition, the optimization of the sampling interval results in an improvement in decision-making performance when the sampling interval is set to be close to the negative autocorrelation time for a small number of slot machines^9^. However, the advantage of the correlation characteristics disappears when the number of slot machines is large.

## Discussion

In this section, we compare the proposed laser-chaos-based method with previous photonic methods for solving the multi-armed bandit problem with a large number of slot machines. Previous photonic methods have used hierarchical structures^10^ and laser networks^15^, in which the decision-making performance was affected by the arrangement of the slot machines. However, in the laser-chaos-based method, all of the slot machines are compared in parallel, and the selection is determined by the maximum chaotic temporal waveform with bias. Thus, the laser-chaos-based method is independent of the arrangement of the slot machines, and is advantageous for solving large-scale multi-armed bandit problems.

The use of chaotic temporal waveforms in semiconductor lasers makes it possible to generate fast random signals in the gigahertz order, which are implemented as physical random number generators^37^. Random signals can be generated easily using a semiconductor laser with optical feedback, and the generation speed is much faster than that of pseudo-random number generators in a computer. In addition, one of the advantages of using chaotic signals is the existence of temporal correlation, which can be useful for decision making with a small number of slot machines, e.g., *N* = 2. However, the advantages of correlation disappear as the number of temporal waveforms (slot machines) increases. In addition, we speculate that chaotic signals generated from other nonlinear dynamical systems could also be effective for decision making using the proposed method.

We also demonstrated that a smaller scaling exponent (γ = 0.97) is obtained using laser-chaos-based decision making, compared with well-known software algorithms. However, the difference in the scaling exponents is very small, and we consider the scaling performance of the laser-chaos-based method to be comparable to that of well-known software algorithms. One of the advantages of chaotic temporal waveforms is their generation speed, which is several gigahertz; thus, fast decision making can be achieved using chaotic temporal waveforms generated by semiconductor lasers.

In the proposed method, white Gaussian noise could be utilized instead of chaotic temporal waveforms. In particular, we found that the difference in the CDRs obtained from white Gaussian noise and chaotic temporal waveforms decreases with an increase in the number of slot machines, unlike the results in the literature^9,10^, because the assigned temporal waveforms show no correlation (i.e., are independent) among them. We speculate that the correlation characteristics and statistical distribution of chaotic temporal waveforms are less important for large numbers of slot machines, and no significant difference is obtained between the schemes using white Gaussian noise and chaotic temporal waveforms. In fact, an advantage of using chaotic temporal waveforms is their generation speed. Fast gigahertz-frequency chaotic oscillations can be utilized as physical random numbers for decision making.

In the proposed method, the number of chaotic temporal waveforms is required to be the same as the number of slot machines *N*. However, chaotic temporal waveforms can be generated from a smaller number of semiconductor lasers using time-multiplexing. In other words, a chaotic temporal waveform generated by a semiconductor laser can be divided into multiple temporal waveforms, which are then assigned to multiple slot machines for decision making. In this case, it is important to reduce the cross-correlation among the divided temporal waveforms to generate independent chaotic temporal waveforms. In addition, there is a trade-off between the generation speed of the chaotic temporal waveforms and the number of semiconductor lasers used for decision making.

In this study, we solved the multi-armed bandit problem with pre-defined hit probabilities. However, we did not attempt to solve the multi-armed bandit problem with hit probabilities that are defined based on a statistical distribution^38^; this decision-making setup will be the focus of our future work.

## Conclusions

We numerically investigated a decision-making method for solving the multi-armed bandit problem using bias control of chaotic temporal waveforms of laser intensities in a semiconductor laser with optical feedback. Each chaotic temporal waveform was assigned to a slot machine with an unknown hit probability. Chaotic temporal waveforms were sampled, and the amplitudes of these temporal waveforms with biases were compared. The slot machine assigned to the temporal waveform with the maximum amplitude was selected. The amplitude of the chaotic temporal waveform was controlled by adding or subtracting the bias based on the results of slot machine selection using the tug-of-war method. We achieved successful decision making for the multi-armed bandit problem with up to 1024 slot machines. We also investigated the scaling characteristics of the decision-making performance as the number of slot machines increased. We identified a power-law relationship between the number of plays required for correct decision making and the number of slot machines. The scaling exponent was 0.97, which is close to one and better than those reported in previous studies. We compared the laser-chaos-based method with well-known software algorithms (*ε*-greedy, softmax, UCB1-tuned, and Thompson sampling), and demonstrated that the laser-chaos-based method outperformed them. Finally, we investigated the effect of negative and positive correlations of chaotic temporal waveforms on decision-making performance, and found that negative-correlated temporal waveforms outperformed positive-correlated and independent temporal waveforms for the two-armed bandit problem within a certain parameter range. The laser-chaos-based method is a promising approach to decision making for large-scale multi-armed bandit problems. This method can also be applied for adaptive channel selection in wireless and optical communications using photonic accelerators.

## Methods

### Numerical model of a semiconductor laser with optical feedback

We numerically generate chaotic temporal waveforms in a semiconductor laser with optical feedback using the Lang–Kobayashi equations^25–27^. These equations are described as follows:

7 $$\frac{dE\left(t\right)}{dt}=\frac{1+i\alpha }{2}\left[\frac{{G}_{N}\left(N\left(t\right)-{N}_{0}\right)}{1+\varepsilon {\left|E\left(t\right)\right|}^{2}}-\frac{1}{{\tau }_{p}}\right]E\left(t\right)+\kappa E\left(t-\tau \right)\text{exp}\left(-i\omega \tau \right),$$

8 $$\frac{dN\left(t\right)}{dt}=J-\frac{N\left(t\right)}{{\tau }_{s}}-\frac{{G}_{N}\left(N\left(t\right)-{N}_{0}\right)}{1+\varepsilon {\left|E\left(t\right)\right|}^{2}}{\left|E\left(t\right)\right|}^{2},$$

where $E(t)$ and $N(t)$ represent the complex electric-field amplitude and carrier density of the semiconductor laser with optical feedback, respectively. The parameters and their values are summarized in Table 1^39^.

**Table 1:** Parameter values used in the numerical simulations of a semiconductor laser with optical feedback.

Symbol	Parameter	Value
*G*_*N*_	Gain coefficient	8.40 × 10^−13^ m^3^ s^−1^
N_0_	Carrier density at transparency	1.40 × 10^24^ m^−3^
τ_*p*_	Photon lifetime	1.927 × 10^−12^ s
τ_*s*_	Carrier lifetime	2.04 × 10^−9^ s
τ_*in*_	Round-trip time in internal cavity	8.0 × 10^−12^ s
α	Linewidth enhancement factor	3.0
ε	Gain saturation coefficient	2.5 × 10^−23^
*r*_2_	Reflectivity of laser facet	0.556
*r*_3_	Reflectivity of external mirror	0.036
κ = (1 − *r*_2_^2^)*r*_3_/(*r*_2_τ_*in*_)	Optical feedback strength	5.592 × 10^9^ s^−1^
*J/J*_*th*_	Normalized injection current	1.36
*c*	Speed of light	2.998 × 10^8^ m s^−1^
*L*	External cavity length	0.3 m
τ = 2*L*/*c*	Round-trip time of light in external cavity	2.001 × 10^−9^ s
λ	Optical wavelength	1.537 × 10^−6^ m
ω = 2π*c*/λ	Optical angular frequency	1.226 × 10^15^ s^−1^
*N*_*th*_ = N_0_ + 1/(*G*_*N*_τ_*p*_)	Carrier density at lasing threshold	2.018 × 10^24^ m^−3^
*J*_*th*_ = *N*_*th*_/τ_*s*_	Injection current at lasing threshold	9.891 × 10^32^ m^−3^ s^−1^

### Chaotic temporal waveforms

**Figure 6:**
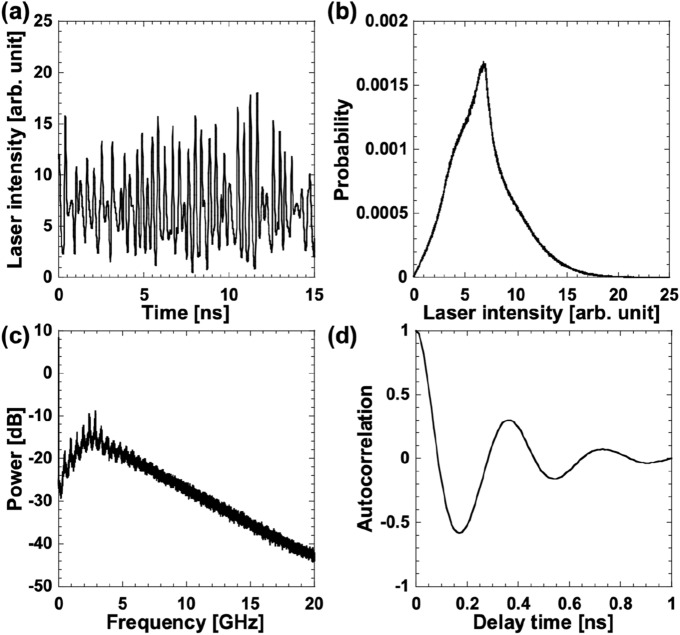
Chaotic temporal waveforms for decision-making. Examples of chaotic temporal waveform of (**a**) laser intensity, (**b**) histogram, (**c**) fast Fourier transform (FFT), and (**d**) autocorrelation function.

We numerically calculated the chaotic temporal waveforms of a semiconductor laser with optical feedback for decision making. Figure 6a shows an example of the chaotic temporal waveforms generated from the Lang–Kobayashi equations. The temporal waveform fluctuates chaotically with an order of nanoseconds. Figure 6b shows a histogram of the chaotic temporal waveforms for the laser intensities demonstrated in Fig. 6a. The histogram displays a Gaussian-like distribution; however, the distribution is skewed at larger intensities. Figure 6c shows fast Fourier transform (FFT) of the chaotic temporal waveform illustrated in Fig. 6a. The FFT is widely distributed, and the peak frequency of the FFT corresponds to 2.9 GHz. Figure 6d shows the autocorrelation function of the chaotic temporal waveform. The second peak of the cross-correlation value is 0.35 ns, corresponding to the inverse of the peak frequency (2.9 GHz) of the FFT in Fig. 6c. Other independent chaotic temporal waveforms are generated from different initial conditions and used for decision making.

### Optimization of bias coefficient

**Figure 7:**
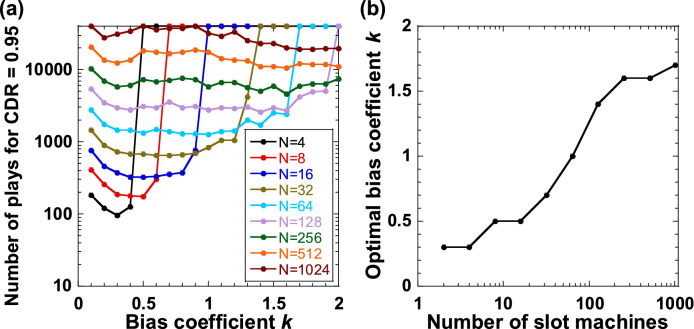
Optimization of bias coefficient *k* for different numbers of slot machines *N*. (**a**) Number of plays to achieve CDR = 0.95 as the bias coefficient *k* increases for different values of *N*. (**b**) Optimal bias coefficient *k* for different values of *N* obtained from (**a**).

We optimized the bias coefficient *k* for different numbers of slot machines *N* to obtain the CDR and scaling characteristics shown in Fig. 3. Figure 7a shows the number of plays required to achieve a CDR of 0.95 when *k* is changed for different values of *N*. The optimal *k* is obtained from the minimum number of plays required to achieve a CDR of 0.95, as shown in Fig. 7a. Figure 7b shows the optimal *k* for different values of *N,* obtained from Fig. 7a. The optimal value of *k* increases monotonically as *N* increases, indicating that a large *k* is required for a large *N*. A small *k* results in too much exploration when *N* is large, so *k* must be sufficiently large for a large *N*. These optimal values of *k* are used to obtain the CDR curve and scaling characteristics shown in Fig. 3.

## Data Availability

The datasets generated during the current study are available from the corresponding author upon reasonable request.
